# The Complete Chloroplast Genome of *Carya cathayensis* and Phylogenetic Analysis

**DOI:** 10.3390/genes13020369

**Published:** 2022-02-18

**Authors:** Jianshuang Shen, Xueqin Li, Xia Chen, Xiaoling Huang, Songheng Jin

**Affiliations:** 1 Jiyang College, Zhejiang A&F University, Zhuji 311800, China; shenjianshuang18@163.com (J.S.); lxqin@zafu.edu.cn (X.L.); cx2912@zafu.edu.cn (X.C.); jyxyhxl@aliyun.com (X.H.); 2 State Key Laboratory of Subtropical Silviculture, School of Forestry and Biotechnology, Zhejiang A&F University, Hangzhou 311300, China

**Keywords:** *Carya cathayensis*, chloroplast genome, genome skimming, phylogenetic relationship

## Abstract

*Carya cathayensis*, an important economic nut tree, is narrowly endemic to eastern China in the wild. The complete cp genome of *C. cathayensis* was sequenced with NGS using an Illumina HiSeq2500, analyzed, and compared to its closely related species. The cp genome is 160,825 bp in length with an overall GC content of 36.13%, presenting a quadripartite structure comprising a large single copy (LSC; 90,115 bp), a small single copy (SSC; 18,760 bp), and a pair of inverted repeats (IRs; 25,975 bp). The genome contains 129 genes, including 84 protein-coding genes, 37 tRNA genes, and 8 rRNA genes. A total of 252 simple sequence repeats (SSRs) and 55 long repeats were identified. Gene selective pressure analysis showed that seven genes (*rps15*, *rpoA*, *rpoB*, *petD*, *ccsA*, *atpI*, and *ycf1-2*) were possibly under positive selection compared with the other *Juglandaceae* species. Phylogenetic relationships of 46 species inferred that *Juglandaceae* is monophyletic, and that *C. cathayensis* is sister to *Carya kweichowensis* and *Carya illinoinensis*. The genome comparison revealed that there is a wide variability of the junction sites, and there is higher divergence in the noncoding regions than in coding regions. These results suggest a great potential in phylogenetic research. The newly characterized cp genome of *C. cathayensis* provides valuable information for further studies of this economically important species.

## 1. Introduction

The genus *Carya*, belonging to the family *Juglandaceae*, comprises ~18 species and 4 varieties, which are distributed in the temperate and tropical regions of East Asia and eastern North America [1,2]. *Carya* species from East Asia and eastern North America are phylogenetically separated [2], while the relationships among some taxa within the genus have not been resolved yet.

Nuclear and plastid DNAs are the basics for phylogenetic reconstruction; the single- or low-copy nuclear genes are most suitable for systematic analyses [3]. Until now, several plastid (*matK, rbcL-atpB, rpoC1, rps16, trnH-psbA*, and *trnL-F*) and nuclear (ITS and *phyA*) DNA markers have been used for the phylogenetic study of the genus *Carya*. These nuclear genes were identified by ortholog screening, cloning, and sequencing; however, these methods can be costly and time-consuming. Compared with the nuclear genome, the chloroplast (cp) genome is an excellent alternative owing to its small size (75–250 Kb) [4], easily obtainable sequences by the low-cost next-generation sequencing (NGS) technique, and less interference from homologous regions. Besides the genic regions, the noncoding regions of cp genomes can also be harnessed for phylogenetic analysis due to a relatively high level of genetic variation resulting from the low selective pressure [5]. In addition, structural rearrangements, such as the loss of introns, genes, or even inverted repeats, extensively occur in the plastid genomes of many flowering plants [6,7,8,9,10,11]. Recently, the cp genomes of *C kweichowensis* [12], *C. cathayensis* [13], and *C. illinoinensis* (NBCI accession number: NC_041449.1) have been published, and the publication of more cp genomes of *Carya* species will facilitate the identification of genetic variations via sequence comparison, providing new insights into the evolutionary history and interspecific relationships among *Carya* species.

*C. cathayensis* (Chinese hickory) is naturally distributed in moist valleys at altitudes of 500–1200 m in Zhejiang, Jiangxi, and Anhui Provinces, China. Because of its high nutritional and economic values, *C. cathayensis* has been widely cultivated in Zhejiang Province, China [14]. *C. cathayensis* is an important economic nut tree and is vulnerable to abiotic factors [15,16], suggesting that suitable habitat is essential for its survival in the wild. In recent years, with the changes in climate and over-exploitation, the conservation of wild *C. cathayensis* populations has become an urgent task. The nuclear genome and cp genome of *C. cathayensis* have been released [13,17], although the cp genome has not been reported in detail. The cp genome of *C. cathayensis* is essential for the development of conservation and breeding strategies.

In this study, we present the whole plastome sequence of *C. cathayensis* and explore the utility of this new genomic resource and relationship with that of other *Carya* species. These results will lay the foundation for future phylogenetic and structural diversity studies of *Carya*.

## 2. Materials and Methods

### 2.1. DNA Extraction, Sequencing, and cp Genome Assembly

The young green leaves of *C. cathayensis* were collected from the nursery of Zhejiang A&F University (stored in the Institute of Botany, Chinese Academy of Sciences Mem, and the specimen accession number is PE00820836) and stored immediately at −80 ℃. Total genomic DNA was isolated from the leaves using a modified CTAB method [18]. After ensuring the quality of DNA, shotgun libraries (250 bp) were constructed in accordance with the standard protocol suggested by the manufacturer’s instructions (Illumina Inc., San Diego, CA, USA). Sequencing was performed with an Illumina Hiseq 2500 platform (Genepioneer Biotechnologies Co., Ltd.; Nanjing, China) with the PE150 strategy.

Quality control for the raw sequencing data was carried out using the package FastQC (version 0.11.8. Available online: http://www.bioinformatics.babraham.ac.uk/proje-cts/fastqc/, accessed on 8 September 2021). High-quality clean reads were obtained by removing the adapters and low-quality reads from the raw data using Trimmomatic (version 0.35) [19]. The *C. cathayensis* cp genome was assembled using the SPAdes pipeline [20] with the *Cyclocarya paliurus* cp genome as the reference (NCBI accession number: NC_034315).

### 2.2. Annotation of the C. cathayensis cp Genome

*C. cathayensis* cp genome annotation was performed via the CpGAVAS pipeline [21]. The annotated *C. cathayensis* genome was deposited to GenBank under accession number MN892516. The circular gene map was visualized in OGDRAWv1.2. Available online: http://ogdraw.mpimp-golm.mpg.de/, accessed on 12 September 2021). Relative synonymous codon usage (RSCU) was determined by CodonW version 1.4.4. Available online: http://codonw.sourceforge.net/, accessed on 15 September 2021).

### 2.3. Identification of Repeats

REPuter [22,23] was used to identify the repeat sequences [24,25] using the parameters reported by [7]. Then, the online microsatellite identification tool (MISA. Available online: https://webblast.ipk-gatersleben.de/misa/, accessed on 21 September 2021) [26] was applied to predict cpSSRs with default parameters.

### 2.4. Phylogenetic Analysis

To determine the phylogenetic relationships among *Juglandaceae* species, a Bayesian inference (BI) tree was inferred using protocols suggested by [27]. An alignment of 46 cp genomic sequences (See in ‘Data Availability Statement’ part) was created using the MAFFT online version [28,29] with default parameters.

### 2.5. Genomic Comparison with Related Species

The online tool Irscope [30] was employed to draw the genetic architecture of the IR/SSC and IR/LSC junctions. mVISTA [31] was used to compare the complete *C. cathayensis* cp genome to that of five related species including *C. kweichowensis*, *C. illioninensis*, *C. paliurus*, *Juglans cathayensis*, and *Platycarya strobilacea*. The shuffle-LAGAN mode was used in mVISTA [31], with the annotation of *Quercus variabilis* as the reference. The sequences were initially aligned using the MAFFT online version [28,29], the pi value of each gene was calculated through alignment of each gene CDS sequence of different species using vcftools, and the ratios of nonsynonymous (*Ka*) to synonymous (*Ks*) substitutions (*Ka*/*Ks*) in protein-coding genes were determined by KaKs_Calculator.

## 3. Results

### 3.1. Genome Features of C. cathayensis

Filtering of the raw sequencing data yielded a total of 12,470,465 clean paired-end reads. There were 3.7 G bases, of which 89.47% of bases had a quality score higher than Q30. The whole cp genome of *C. cathayensis* is 160,825 bp in length, with a GC content of 36.13%. The genome assembly had an average read coverage of higher than 700×. The synteny was identified by comparing the *C. cathayensis* cp genome to the reference (Table S1), which showed that most of the sequences of the genomes were conserved.

**Figure 1:**
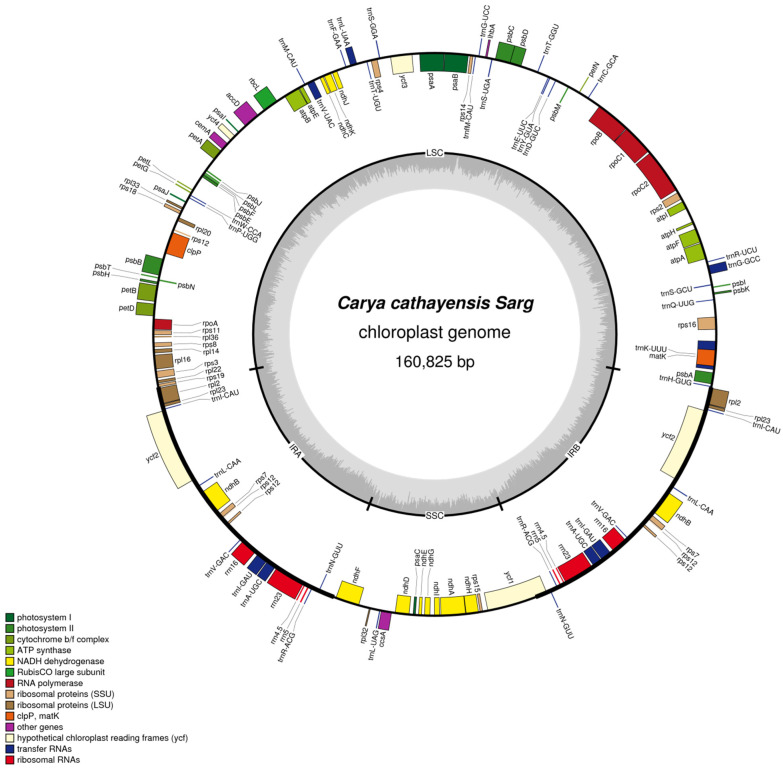
The complete *C. cathayensis* chloroplast (cp) genome. Genes shown outside the outer circle are transcribed clockwise, whereas those shown inside are transcribed counterclockwise. The gray plots in the inner circle represent GC contents. The circular gene map was drawn using OGDRAWv1.2.

**Figure 2:**
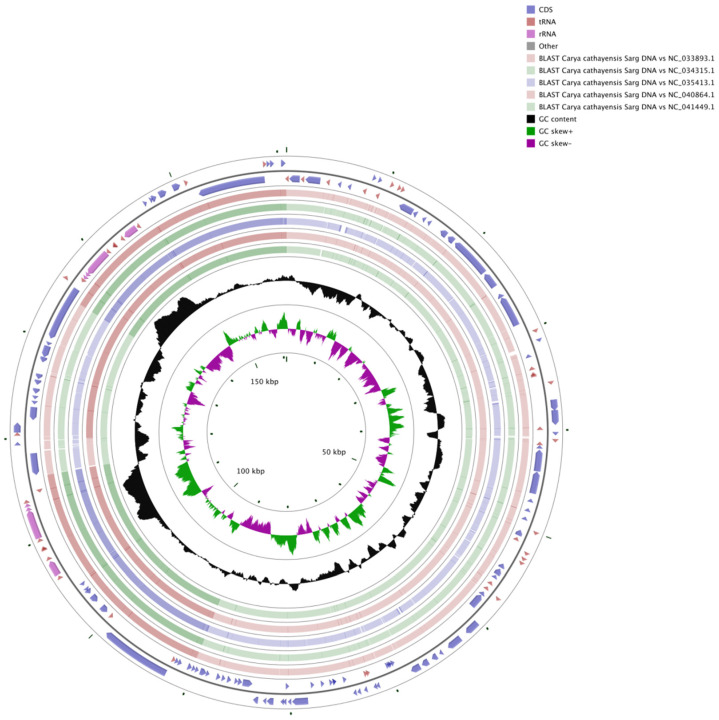
GC content of the *C. cathayensis* cp genome.

The genome of *C. cathayensis* displays a typical quadripartite structure, containing one large single copy (LSC; 90,115 bp) region, one small single copy (SSC; 18,760 bp) region, and two inverted repeat regions (IRs; 25,975 bp each) (Figure 1). The overall GC content is 36.13%. The IR regions have a relatively higher GC content compared with other regions (Figure 2). A total of 129 genes were identified, including 84 protein-coding genes, 37 transfer RNA (tRNA) genes, and 8 ribosomal RNA (rRNA) genes (Table 1). Seventeen genes are duplicated in IRs, including six protein-coding genes (*rps7*, *rps12*, *rpl2*, *rpl23*, *ndhB*, *ycf2*) (Table 1). In total, 18 intron-containing genes (12 protein-coding and 6 tRNA genes) were annotated (Table 2), among which there are only 3 protein-coding genes (*rps12*, *ycf3*, and *clpP*) with 2 introns and the others with 1 intron. Gene *rps12* of *C*. *cathayensis* has its 5′-end exon situated in the LSC region and its 3′-end exons located in the IR region (Figure 1, Table 2).

**Figure 3:**
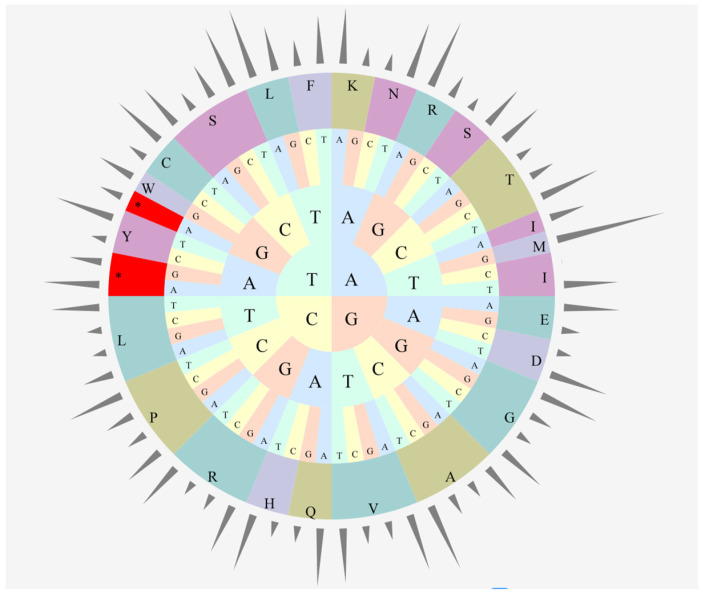
Codon usage frequency of the *C. cathayensis* cp genome.

The relative frequency of synonymous codons of the *C. cathayensis* cp coding sequence was estimated. The results show that all genes are encoded by 26,476 codons, and the 4 most frequently used codons were AUU (isoleucine), AAA (lysine), GAA (glutamic acid), and AAU (asparagine), pertaining to 1145 (4.32%), 1066 (4.03%), 1040 (3.93%), and 1004 (3.79%) codons, respectively (Table S2 and Figure 3). The two most frequently used amino acids were leucine (2780) and isoleucine (2350); cysteine was the least abundant, with only 308 hits. A- and U-ending codons accounted for 70.62% among all codons.

### 3.2. Analysis of Long Repeats and Simple Sequence Repeats (SSRs)

We identified 24 forward, 9 reverse, 3 complement, and 13 palindrome repeats in the cp genome of *C. cathayensis* (Table S3). Most repeats ranged from 20 to 62 bp in length. The longest forward repeat with 62 bp resided in the LSC region. A total of 46, 5, and 4 long repeats were found in the LSC, SSC, and IR regions, respectively. Three forward repeats were found in the two IRs, including one repeat associated with the *rpl14* and *tRNA-UGC* genes, one with the *IGS* genes, and one with the *tRNA-CCA* and *tRNA-GUU* genes.

A total of 252 SSRs were identified in the *C. cathayensis* cp genome (Table S4), among which 199, 12, 64, 2, and 1 were mono-, di-, tri-, tetra-, and pentanucleotide repeats, respectively. Mononucleotide SSRs were the richest (occupied 78.97%), and the mononucleotide A+T repeat units occupied the highest portion (75.00%).

### 3.3. Phylogenetic Analysis

**Figure 4:**
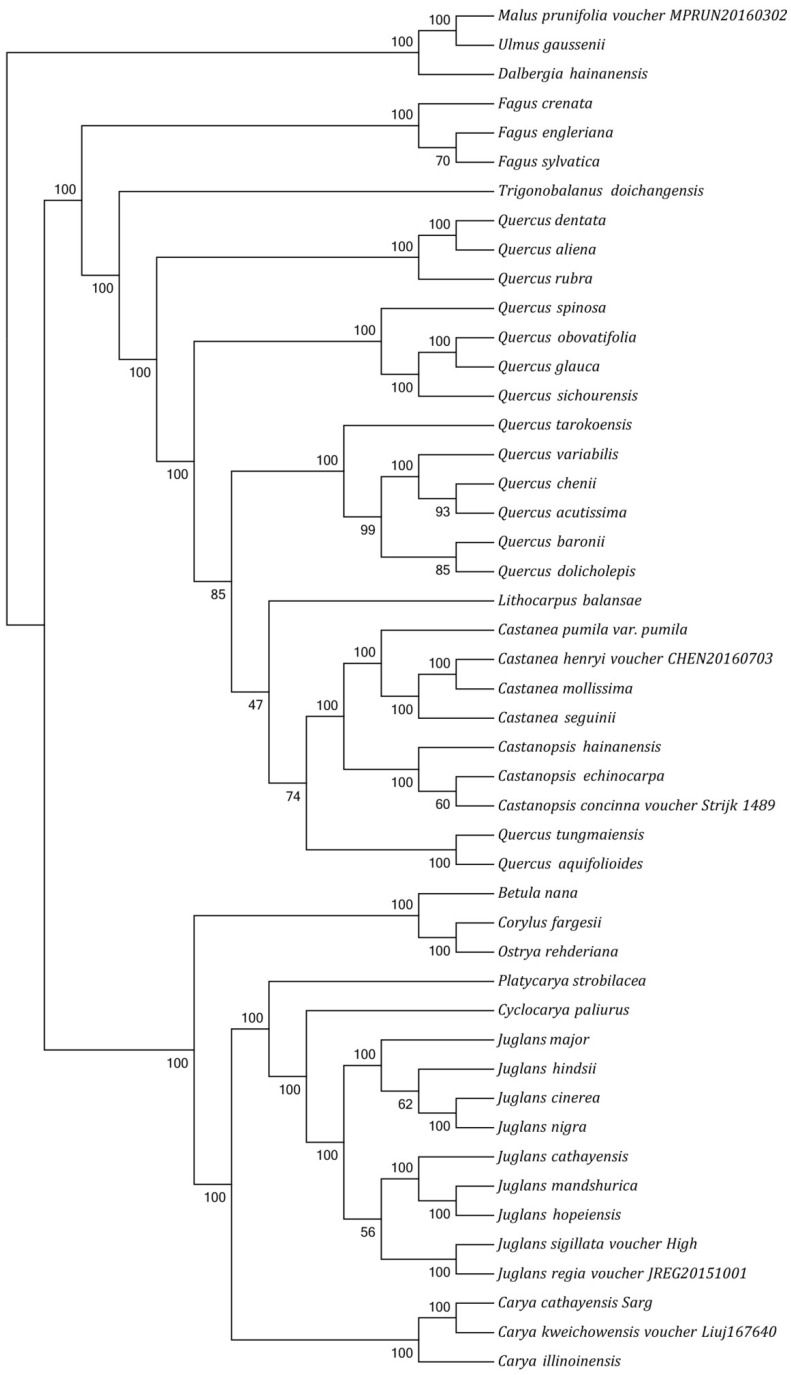
ML phylogenetic tree of 46 complete cp genomes resolved by Raxml. Bootstrap values are shown near each node.

Phylogenetic analysis was carried out based on an alignment of the concatenated nucleotide sequences of all 46 angiosperm cp genomes (Figure 4). MAFFT was employed for multiple sequence alignment. The phylogenetic relationship was reconstructed using the GTR-γ model by RAxML, and *Malus prunifolia*, *Ulmus gaussenii*, and *Dalbergia hainanensis* were used as outgroups. Almost all relationships inferred from the cp genome data based on the maximum likelihood (ML) tree received strong support, with the support values ranging from 47 to 100. In addition, genera *Betula*, *Corylus*, and *Ostrya* were found to be sister to *Juglans*, whereas *Platycarya* and *Cyclocarya* were more closely related to *Juglans* (Figure 4). The well-supported phylogenetic tree (Figure 4) indicates that the genus *Carya* is monophyletic and is most closely related to the cluster formed by another genus of *Juglandaceae*. *C. cathayensis* is sister to *C. kweichowensis*, and they are sister to *C. illinoinensis* successively, with high support scores (bootstrap = 100; Figure 4).

### 3.4. Comparative Analysis of Genome Structure

**Figure 5:**
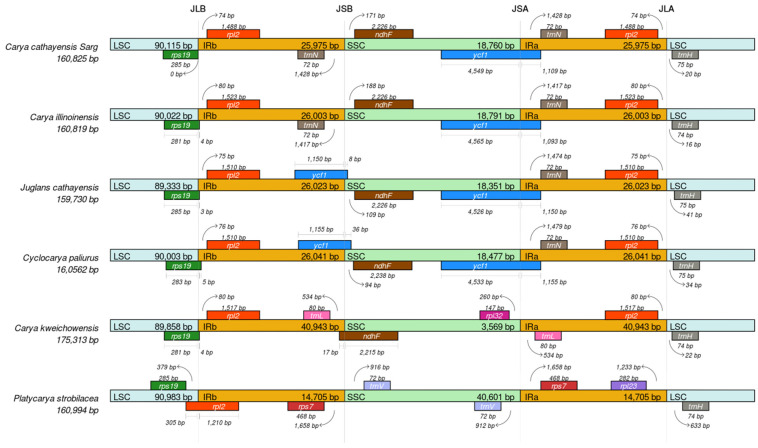
Comparison of the LSC, SSC, and IR regions among six selected cp genomes in the family *Juglandaceae*. Genes are denoted by colored boxes. The gaps between the genes and boundaries are proportional to the distances in bps.

To further resolve the structural evolutionary history of the cp genomes of the genus *Carya*, we compared the IR/SSC and IR/LSC junctions across six selected *Juglandaceae* species, including *C. cathayensis*, *C. illinoinensis*, *C. kweichowensis*, *Platycarya strobilacea*, *Cyclocarya paliurus*, and *Juglans cathayensis*. The results of the IRscope analysis are presented in Figure 5. We observed a wide variability of the junction sites in these cp genomes. For example, in the genus *Carya*, *C. cathayensis* exhibited similar JLB, JSB, and JSA junction sites compared with its elder sister species *C. illinoinensis* (Figure 4 and Figure 5). All species used in this study had an IRa/b region of ~25,900 bp and an SSC region of ~18,700 bp. By contrast, *C. kweichowensis*, which is most closely related to *C. cathayensis* and *C. illioninensis*, displayed an extremely large IRa/b region of 40,943 bp. In addition, the *C. kweichowensis* cp genome showed some striking structural differences compared to its sister species. For example, the *rps19* gene was shifted by 285 bp from the LSC to IRb at the LSC/IRb border, *trnL* was located in the IRa/b regions instead of the SSC region, and *ycf1* was absent from the JSA site. Moreover, we observed variations in the IR/SSC and IR/LSC junction sites across other genera in the family *Juglandaceae* (Figure 5).

**Figure 6:**
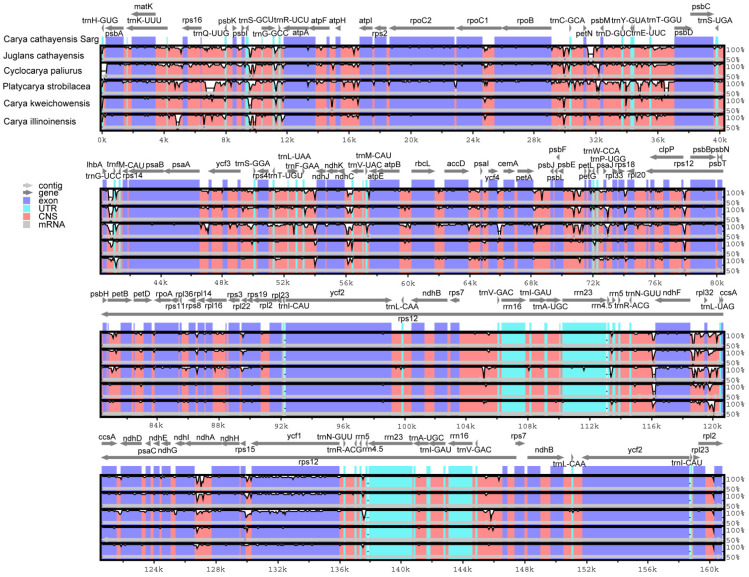
Variable characters in homologous regions among *C. cathayensis* and five related species. The homologous regions are oriented according to their locations in the cp genome. The gray arrows above the alignment indicate the gene orientations. The Y-axis shows the identity from 50% to 100%.

**Figure 7:**
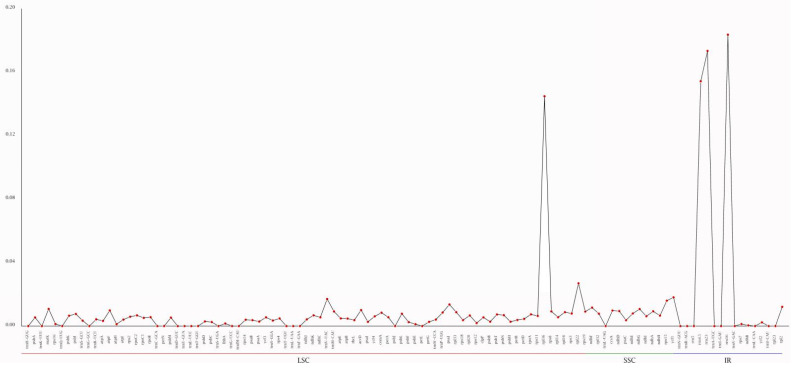
Gene nucleotide variability (pi) values of six *Juglandaceae* species. The Y-axis shows the pi values; the X-axis shows the genes.

A cp genome identity analysis was performed on the six *Juglandaceae* species described above, with the *C. cathayensis* cp genome used as a reference (Figure 6). This analysis found a relatively higher level of divergence in the noncoding than in the coding regions. We also identified a considerable number of variations in the noncoding cp sequences, such as *trnC-GCA*, *trnW-CCA*, *trnI-CAU*, and *trnI-UAG*, of species in the genus *Carya* (Figure 6). Gene nucleotide variability (pi) values of six selected *Juglandaceae* species (including *C. cathayensis*, *C. illinoinensis*, *C. kweichowensis*, *Platycarya strobilacea*, *Cyclocarya paliurus*, and *Juglans cathayensis*) are shown in Figure 7, where the values of *LSC.rpl36*, *IR. rrn4.5*, *rrn23*, and *rrn16* are higher than 1, while the values of other genes are lower than 0.03. The results show that there is lower nucleotide diversity among the six *Juglandaceae* species.

To test whether the remaining cp genes in these six species of *Juglandaceae* have undergone selection, the synonymous (*Ks*) and nonsynonymous (*Ka*) substitution rates were calculated (Table S5). The *Ka*/*Ks* ratios were then categorized, with *Ka*/*Ks* < 1, *Ka*/*Ks* = 1, and *Ka*/*Ks* > 1 denoting purifying, neutral, and positive selections, respectively, in the context of a codon substitution model. The results show that only seven genes of *C. cathayensis*, namely, *rps15*, *rpoA*, *rpoB*, *petD*, *ccsA*, *atpI*, and *ycf1-2*, underwent positive selection compared with the other *Juglandaceae* species (Table S4). By contrast, most genes were shown to have undergone purifying selection, which was evidenced by a *Ka*/*Ks* ratio below 1 and the presence of negatively selected sites within some genes.

## 4. Discussion

Plant chloroplast genomes may have 63–209 genes, but most are concentrated between 110 and 130, with a highly conserved composition and arrangement, including photosynthetic genes, chloroplast transcriptional expression-related genes, and some other protein-coding genes [32]. As with other angiosperms, the cp genome of *C. cathayensis* displays a typical quadripartite structure [32,33], including a pair of inverted repeats (IRs; 25,975 bp each), separated by a large single copy (LSC; 90,115 bp) and a small single copy (SSC; 18,760 bp) region (Figure 1). In total, 129 genes, including 84 protein-coding genes, 37 tRNA genes, and 8 rRNA genes, were identified in our study. The overall GC content is 36.13%, which is similar to that observed for other *Carya* species (35.8–36.3%) [12,13,34]. It is obvious that the DNA G + C content of the IR region is higher than that of other regions (LSC, SSC) (Figure 2); this phenomenon is very common in other flowering plants [25,34,35]. GC skewness has been shown to be an indicator of DNA lead chains, lag chains, replication origin, and replication terminals, which is a very important indicator of species affinity [36]. The *rps12* gene of *C*. *cathayensis* has its 5′-end exon situated in the LSC region and its 3′-end exons located in the IR regions (Figure 1); this result is similar to that for the congeneric species *C. sinensis* [34]. However, there is a certain difference with previous reports of the *C*. *cathayensis* cp genome, such as the length (160,666 bp), GC contents (36.2%), and annotated genes (86 protein-coding genes, 39 tRNA genes) of the whole cp genome [13]. The difference may be due to the geographical isolation or evolutionary differences of different plant populations from An’hui and Zhejiang Provinces, which facilitate the identification of genetic variations via sequence comparison, providing new insights into the evolutionary history of *C*. *cathayensis.*

The codon usage bias of cp genomes may be a result of selection and mutation [35]. The frequency of codon usage was estimated for the *C*. *cathayensis* cp genome in this study. We found that all genes are encoded by 26,476 codons, and the 4 most frequently used codons were AUU, AAA, GAA, and AAU; among these codons, A- and U-ending codons are common (Table S2 and Figure 3). This result is similar to the results reported in other angiosperms [6,7,24,37], and these features of codon usage preference can help to better decipher exogenous gene expression and the evolution mechanisms of the cp genome [24,25,38].

The cpSSR markers are excellent tools for phylogenetic research due to several characteristics, including non-recombination, haploidy, uniparental inheritance, and the low substitution rate [39]. They are especially valuable for intraspecific population genetic variation research [40,41] and interspecific evolutionary and identification studies [42,43,44,45,46]. A previous study reported that 213 SSRs and 44 long repeats were identified in the cp genome of *C. illinoinensis* [47], while 252 SSRs and 55 long repeats were identified in our study. This study found mononucleotide SSRs were the richest (occupied 78.97%), and the mononucleotide A+T repeat units occupied the highest portion (75.00%); these results are consistent with a previous study and verify the hypothesis that cpSSRs are generally composed of short polyadenine (polyA) or polythymine (polyT) repeats and rarely contain tandem guanine (G) or cytosine (C) repeats [38,48]. The cpSSRs are mainly distributed in the noncoding regions of the cp genome of *C. cathayensis*; a similar distribution preference of cpSSRs has been reported in other plants, such as *Olea europaea*, *Salviamiltiorrhiza*, and *Avena sativa* [47,49]. Dispersed repeats may facilitate intermolecular recombination and plastome diversity creation, because the genome regions with increased sequence diversity could be formed by repeat sequence abundance in prokarya and eukarya [50]. Hence, these cpSSR markers of *C. cathayensis* could be used to examine the genetic structure, diversity, differentiation, and maternity in *Carya* and provide a new avenue for the development of species protection and preservation strategies.

Phylogenetic analysis was completed on an alignment of all chloroplast genomes from 46 angiosperm species. The well-supported phylogenetic tree (Figure 4) indicates that the genus *Carya* is monophyletic and is most closely related to the cluster formed by another genus of *Juglandaceae*, which is consistent with previous studies [2,12]. The genus *Quercus* was polylogenetic in our analysis, resulting from the embedded branches of the genera *Lithocarpus* and *Castanea*; this result is consistent with previous results [6]. Phylogenetic relationships inferred that *Juglandaceae* is monophyletic, and that *C. cathayensis* is sister to *C. kweichowensis* and *C. illinoinensis* in our study. Previous studies reported that *C. kweichowensis* is one of the representative species of the Asian sect. *Sinocarya*, while *C. illinoinensis* is one of the representative species of the North American sect. *Apocarya* [47]. The *C. cathayensis* used in our study is native to China, in Asia. Thus, we speculated that the above factors led to *C. cathayensis* and *C. kweichowensis* falling into one clade, while *C. cathayensis* and *C. illinoinensis* fell into two clades.

The size variation in angiosperm plastid genomes is often accompanied by the expansion and contraction of the IR and SSC boundary regions [51,52]. It is well known that certain plastome regions show different mutation rates. To further resolve the structural evolutionary history of the cp genomes of the genus *Carya*, we compared the IR/SSC and IR/LSC junctions across six selected *Juglandaceae* species, including *C. cathayensis*, *C. illinoinensis*, *C. kweichowensis*, *Platycarya strobilacea*, *Cyclocarya paliurus*, and *Juglans cathayensis*. We observed a wide variability of the junction sites. The cp genomes of *C. cathayensis* exhibited similar JLB, JSB, and JSA junction sites. We observed variations in the IR/SSC and IR/LSC junction sites across other genera in the family *Juglandaceae*: for example, the *rps19* gene was shifted by 285 bp from the LSC to IRb at the LSC/IRb border, *trnL* was located in the IRa/b regions instead of the SSC region, and *ycf1* was absent from the JSA site (Figure 5). The LSC/IR and SSC/IR borders are relatively conserved among angiosperm plastomes, mostly positioned within *rps19* or *ycf1* [53]. Significant expansions have been reported in other plants, such as in *Pelargonium* × *hortorum* L.H. Bailey [54], *Jasminum nudiflorum* Lindl [55], and *Avena sativa* [49].

This study revealed a relatively higher level of divergence in the noncoding than in the coding regions, similar to what has been reported for the genus *Quercus* from the family *Fagaceae* [6], which is related to the family *Juglandacea*. We also identified a considerable number of variations in the noncoding cp sequences, such as *trnC-GCA*, *trnW-CCA*, *trnI-CAU*, and *trnI-UAG*, of species in the genus *Carya* (Figure 6). Hence, these noncoding sites may be useful for resolving the suspending phylogenetic relationships of *Carya* species [2]. Gene nucleotide variability (pi) values of *LSC.rpl36*, *IR. rrn4.5*, *rrn23*, and *rrn16* were higher than 1, while the values of other genes were lower than 0.03. The results show that there is lower nucleotide diversity among the six *Juglandaceae* species. The results can provide reference for plastome marker selection, which should be carried out based on appropriate evolutionary rates (pi values) [49]. The plastid genome is typically conserved across most angiosperms [55]. Our results found that seven genes (*rps15*, *rpoA*, *rpoB*, *petD*, *ccsA*, *atpI*, and *ycf1-2*) of *C. cathayensis* underwent positive selection (Table S4); other genes were shown to have undergone purifying selection. These results indicate that there is selective pressure on plastid function, where genes encoding proteins for DNA maintenance underwent positive selection, and expression may be relaxed [49].

## 5. Conclusions

The diversification of *C. cathayensis* plastomes is explained by the presence of highly diverse genes, LSC intermolecular recombination, and the co-occurrence of tandem repeats. This study demonstrates that there is a wide variability of the junction sites between the cp genomes of six *Juglandaceae* species, and there is higher divergence in the noncoding regions than in coding regions in the cp genome of *C. cathayensis*. The genus *Quercus* was polylogenetic, resulting from the embedded branches of the genera *Lithocarpus* and *Castanea*. The characterization of the *C. catayensis* cp genome provides valuable genetic information for the phylogenetic study and the development of conservation strategies of the genus *Carya*.

## Figures and Tables

**Table 1:** Annotated genes in the *C. cathayensis* cp genome.

Category	Group of Genes	Name of Gene
Self-replication	Ribosomal RNA	*rrn4.5* ^3^, *rrn5* ^3^, *rrn16* ^3^, *rrn23* ^3^
	Transfer RNA	*trnY-GUA*, *trnW-CCA*, *trnV-UAC* ^1^, *trnV-GAC* ^3^, *trnT-UGU*, *trnT-GGU*, *trnS-UGA*, *trnS-GGA*, *trnS-GCU*, *trnR-UCU*, *trnR-ACG* ^3^, *trnQ-UUG*, *trnP-UGG*, *trnN-GUU* ^3^, *trnM-CAU*, *trnL-UAG*, *trnL-UAA* ^1^, *trnL-CAA* ^3^, *trnK-UUU* ^1^, *trnI-GAU* ^1,3^, *trnI-CAU* ^3^, *trnH-GUG*, *trnG-UCC*, *trnG-GCC* ^1^, *trnfM-CAU* ^4^, *trnF-GAA*, *trnE-UUC*, *trnD-GUC*, *trnC-GCA*, *RNA-UGC* ^1,3^
	Small subunit of ribosome	*rps2*, *rps3*, *rps4*, *rps7* ^3^, *rps8*, *rps11*, *rps12* ^2,3^, *rps14*, *rps15*, *rps16* ^1^, *rps18*, *rps19*
	Large subunit of ribosome	*rpl2* ^1,3^, *rpl14*, *rpl16* ^1^, *rpl20*, *rpl22*, *rpl23* ^3^, *rpl32*, *rpl33*, *rpl36*
	RNA polymerase subunits	*rpoA*, *rpoB*, *rpoC1* ^1^, *rpoC2*
	Subunits of photosystem I	*psaA*, *psaB*, *psaC*, *psaI*, *psaJ*
	Subunits of photosystem II	*psbA*, *psbB*, *psbC*, *psbD*, *psbE*, *psbF*, *psbH*, *psbI*, *psbJ*, *psbK*, *psbL*, *psbM*, *psbN*, *psbT*
Photosynthesis	Subunits of cytochrome	*petA*, *petB* ^1^, *petD* ^1^, *petG*, *petL*, *petN*
	Subunits of ATP synthase	*atpA*, *atpB*, *atpE*, *atpF* ^1^, *atpH*, *atpI*
	Large subunit of RuBisCO	*rbcL*
	Subunits of NADH	*ndhA* ^1^, *ndhB* ^1,3^, *ndhC*, *ndhD*, *ndhE*, *ndhF*, *ndhG*, *ndhH*, *ndhI*, *ndhJ*, *ndhK*
Other gene	Maturase	*matK*
	Envelope membrane protein	*cemA*
	Subunit of acetyl-CoA	*accD*
	C-type cytochrome synthesis gene	*ccsA*
	Protease	*clpP* ^2^
Unknown function	Conserved open reading frames	*ycf1*, *ycf2* ^3^, *ycf3* ^2^, *ycf4*, *ihbA*

^1^ Gene containing a single intron; ^2^ gene containing two introns; ^3^ two gene copies in the IRs; ^4^ duplicated gene in the LSC region.

**Table 2:** Genes with introns in the *C. cathayensis* cp genome.

Gene	Region (bp)	Exon Ⅰ (bp)	Intron Ⅰ (bp)	Exon Ⅱ (bp)	Intron Ⅱ (bp)	Exon Ⅲ (bp)
*atpF*	LSC	144 ^—^	762	411 ^—^		
*clpP*	LSC	71 ^—^	847	292 ^—^	617	227 ^—^
*ndhA*	SSC	552 ^—^	1211	540 ^—^		
*ndhB*	IRB	777 ^—^	686	762 ^—^		
*ndhB*	IRA	775 ^+^	686	760 ^+^		
*petB*	LSC	4 ^+^	822	640 ^+^		
*petD*	LSC	6 ^+^	615	485 ^+^		
*rpl16*	LSC	9 ^—^	919	399 ^—^		
*rpl2*	IRB	390 ^—^	663	435 ^—^		
*rpl2*	IRA	388 ^+^	663	433 ^+^		
*rpoC1*	LSC	430 ^—^	843	1619 ^—^		
*rps12*	IRB	114 ^—^	-	229 ^+^	537	29 ^+^
*rps12*	IRA	114 ^—^	-	231 ^—^	537	29 ^—^
*rps16*	LSC	40 ^—^	894	230 ^—^		
*trnA-UGC*	IRB	36 ^+^	801	40 ^+^		
*trnA-UGC*	IRA	38 ^—^	801	42 ^—^		
*trnG-GCC*	LSC	22 ^+^	715	45 ^+^		
*trnI-GAU*	IRB	40 ^+^	950	33 ^+^		
*trnI-GAU*	IRA	42 ^—^	950	35 ^—^		
*trnK-UUU*	LSC	37 ^—^	2557	35 ^—^		
*trnL-UAA*	LSC	35 ^+^	524	48 ^+^		
*trnV-UAC*	LSC	38 ^—^	615	37 ^—^		
*ycf3*	LSC	126 ^—^	720	229 ^—^	793	151 ^—^

^+^ Exon is transcribed counterclockwise in Figure 1; ^—^ exon is transcribed clockwise in Figure 1; - spliceosomal intron.

## Data Availability

The data used in our study have been submitted to NCBI GenBank (accession number: MN892516). The related species and their GenBank accession numbers (website: https://www.ncbi.nlm.nih.gov/, accessed on 11 October 2021) in this study are listed as follows: *Betula nana* (KX703002), *Castanopsis concinna* (NC_033409), *C. echinocarpa* (NC_023801), *C. hainanensis*(NC_037389), *Castanea henryi* (NC_033881), *C. mollissima* (KY951992), *C. pumila* (KM360048), *C. seguinii* (NC_039749), *Dalbergia hainanensis* (NC_036961), *Fagus crenata* (NC_041252), *F. engleriana* (NC_036929), *F. sylvatica* (NC_041437), *Juglans major* (NC_035966), *J. hindsii* (NC_035965), *J. cinereal* (NC_035960), *J. nigra* (NC_035967), *J. cathayensis* (MF167457), *J. mandshurica* (MF167461), *J. sigillata* (MF167465), *J. hopeiensis* (NC_033894), *J. regia* (NC_028617), *Lithocarpus balansae* (NC_026577), *Malus prunifolia* (NC_031163), *C. illinoinensis* (NC_041449), *C. kweichowensis* (NC_040864), *Cyclocarya paliurus* (NC_034315), *Platycarya strobilacea* (NC_035413), *Quercus acutissima* (NC_039429), *Q. aliena* (NC_026790), *Q. baronii* (NC_029490), *Q. chenii* (NC_039428), *Q. dentata* (NC_039725), *Q. dolicholepis* (KU240010), *Q. obovatifolia* (NC_039972), *Quercus rubra* (JX970937), *Q. sichourensis* (NC_036941), *Q. spinosa* (NC_026790), *Q. tarokoensis* (NC_036370), *Q. variabilis* (KU240009), *Trigonobalanus doichangensis* (NC_023959), and *Ulmus gaussenii* (NC_037840).

## Supplementary Materials

The following supporting information can be downloaded at: https://www.mdpi.com/article/10.3390/genes13020369/s1, Table S1: Statistics of the synteny between the *C. cathayensis* and *Cyclocarya paliurus* cp genomes; Table S2: Codon usage of *C. cathayensis* cp genome from RSCU tools; Table S3: Long repeat sequences in the *C. cathayensis* cp genome; Table S4: Simple sequence repeats (SSR) in the *C. cathayensis* cp genome; Table S5: Ka/Ks ratios of the cp genes from *C. cathayensis* and five related species.

## Abbreviations

LSC: large single copy; SSC: small single copy; IRs: inverted repeats; SSRs: simple sequence repeats; *Ks*: synonymous; *Ka*: nonsynonymous; pi: gene nucleotide variability; RSCU: relative synonymous codon usage.
